# Preliminary therapeutic outcomes of using direct oral anticoagulants to treat venous thromboembolism in gynecological cancer patients

**DOI:** 10.20407/fmj.2018-012

**Published:** 2019-04-17

**Authors:** Sayaka Osaki, Satoshi Kawai, Mayuko Ito, Sayaka Otani, Ryoko Ichikawa, Yutaka Torii, Hiroshi Takahashi, Hiroshi Toyama, Yukio Ozaki, Takuma Fujii

**Affiliations:** 1 Department of Obstetrics and Gynecology, Fujita Health University, School of Medicine, Toyoake, Aichi, Japan; 2 Faculty of Rehabilitation, Fujita Health University, School of Health Sciences, Toyoake, Aichi, Japan; 3 Department of Radiology, Fujita Health University, School of Medicine, Toyoake, Aichi, Japan; 4 Department of Cardiology, Fujita Health University, School of Medicine, Toyoake, Aichi, Japan

**Keywords:** Direct oral anticoagulants, Gynecological cancer, Venous thromboembolism

## Abstract

**Objectives:**

Venous thromboembolism (VTE) is often a problematic complication in patients with gynecological cancer. Despite increasing opportunities to use direct oral anticoagulants (DOACs) to treat VTE, there are no reports on the therapeutic outcomes of DOACs in patients with gynecological cancer; however, there are some studies on cancer patients in general. We retrospectively examined the efficacy and safety of using DOACs to treat VTE in such patients.

**Methods:**

The study cohort comprised 43 patients with gynecological cancer and VTE who received treatment between May 2005 and April 2016. They were divided into two groups: DOACs used (DOAC group, n=21) and only unfractionated heparin (UFH) and warfarin used (standard group, n=22). The rates of improvement and recurrence of VTE and incidence of adverse events were compared between these groups.

**Results:**

At 6 months, the VTE of 85% of patients in the DOAC group and of 75% in the standard group had improved (*p*=0.59). No recurrences of VTE occurred in the DOAC group; where VTE recurred in 12.5% of patients in the standard group. Adverse events occurred in three patients in the DOAC group (15.3%) and one in the standard group (7.7%). Chemotherapy significantly impacted improvement in VTE (*p*=0.01).

**Conclusions:**

Rates of VTE improvement and of recurrence of VTE and adverse events did not differ significantly between the study groups.

## Introduction

Venous thromboembolism (VTE) is the generic name for deep vein thrombosis and pulmonary embolism. VTE can lead to acute and chronic disturbances in pulmonary circulation.^1^ Various factors are known to contribute to occurrence of VTE, including race, underlying disease, lifestyle, physique, and genetic predisposition.^2–4^ Surgery and the presence of malignant tumors are often associated with occurrence of VTE, 15%–40% of gynecological surgeries reportedly resulting in VTE.^2^ VTE is a particularly common complication of gynecological cancer and VTE is often encountered in pathological specimens of gynecological cancer in clinical settings.^5,6^

Currently, low-molecular weight heparin (LMVH) is commonly used to treat and manage VTE in patients with cancer.^7^ However, because there is no reimbursement for LMWH in Japan, unfractionated heparin (UFH) is mainly used in that country. In contrast, opportunities to use direct oral anticoagulants (DOACs) to treat VTE have recently increased, with demonstrations of their efficacy and safety in patients with cancer and VTE.^8,9^ Health insurance reimbursement has been available for DOACs as a treatment for VTE since September 2014 in Japan. We have now also started using DOACs to treat VTE in patients with gynecological cancer. Although there are some studies on the therapeutic outcomes of using DOACs to treat VTE with cancer, no studies have investigated the therapeutic outcomes in patients with gynecological cancer. Therefore, we retrospectively examined the efficacy and safety of using DOACs to treat VTE in such patients.

## Methods

**Figure 1:**
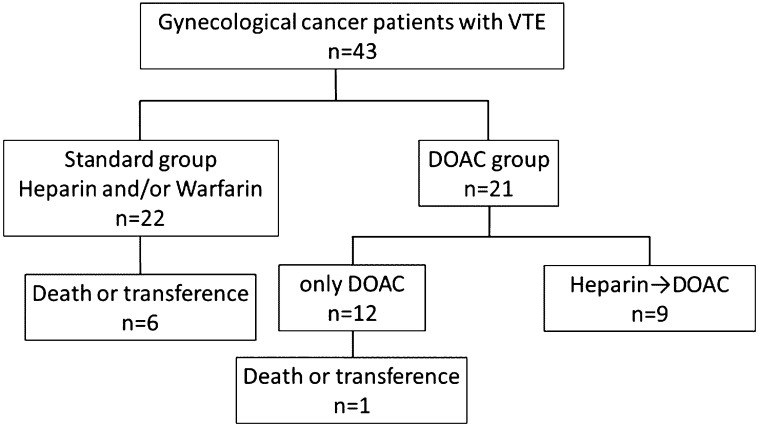
Characteristics of the 43 study patients The DOAC group included 21 patients and the standard group 22s. Nine of the 21 patients in the DOAC group received UFH and/or warfarin.

We examined 43 patients with gynecological cancer and VTE who received treatment at our hospital between May 2005 and April 2016. These patients were divided into a DOAC group (n=21), comprising 12 patients who received only DOACs and nine who received a combination of DOACs, UFH, and warfarin, and a standard group (n=22), comprising patients who received only UFH and warfarin (Figure 1). Eighteen of the 21 patients who were given DOACs received edoxaban (median dose: 30 mg), two rivaroxaban (15 mg dose in each), and one apixaban (15 mg). In the standard group, UFH and warfarin were administered in median doses of 10,000 IU and 2 mg, respectively, the median APTT (activated partial thromboplastin time; APTT) being 48 seconds. APTT was adjusted to 1.72 times the value before treatment. The UFH administered to all patients in the standard group was unfractionated. The patients’ gynecological cancers consisted of 20 ovarian cancers, 11 uterine cancers, five cervical cancers, five peritoneal cancers, and two uterine carcinosarcomas.

VTE was evaluated by ultrasound of the lower limbs or contrast-enhanced computed tomography before and after starting treatment. VTE that had completely resolved or reduced in size on CT or ultrasound images was defined as an “improvement,” whereas VTE that had worsened or recurred after improving was defined as a “recurrence.” The efficacy and safety of VTE treatment using DOACs were retrospectively evaluated on the basis of rates of improvement and recurrence and incidence of adverse events in the 6 months after starting VTE treatment. In was a retrospective study, seven of 43 patients could not be evaluated because of death or transfer to another institution; thus, therapeutic outcomes were evaluated in 20 patients in the DOAC group and 16 in the standard group. Improvement in VTE was observed in 17 patients (85%) in the DOAC group and 12 (75%) in the standard group. No recurrence of VTE was observed in the DOAC group (0%), whereas two patients (12.5%) developed recurrences in the standard group. Adverse events were observed in three patients (15.6%) in the DOAC group and one (7.7%) in the standard group. Any undesirable signs or symptoms, such as bleeding and hepatic dysfunction, were defined as adverse events.

Data were analyzed using IBM SPSS Statistics ver. 22 (IBM Japan). Normally distributed data are presented as means±standard deviation, whereas non-normally distributed data are presented as medians (interquartile range). To compare the two groups, *t*-tests or the Mann–Whitney U test was used for continuous variables, whereas χ^2^ tests were used for categorical variables. The rates of VTE improvement, recurrence, and adverse events were estimated using Kaplan–Meier curves, and each value was compared using the log-rank test. The Cox proportional hazards models was used to calculate the hazard ratios for each endpoint of baseline characteristics. A *p*-value of less than 0.05 was considered to indicate a significant difference. The Institutional Review Board of our hospital approved this study.

## Results

Baseline characteristics according to study group are presented in Table 1. No differences in baseline characteristics were identified between the two groups, nor were there differences between them in primary site, cancer stage at diagnosis, or type of treatment. No association was found between cancer stage and site of VTE. The median follow-up period was 240 days in both groups, with no significant difference (*p*=0.92).

**Figure 2:**
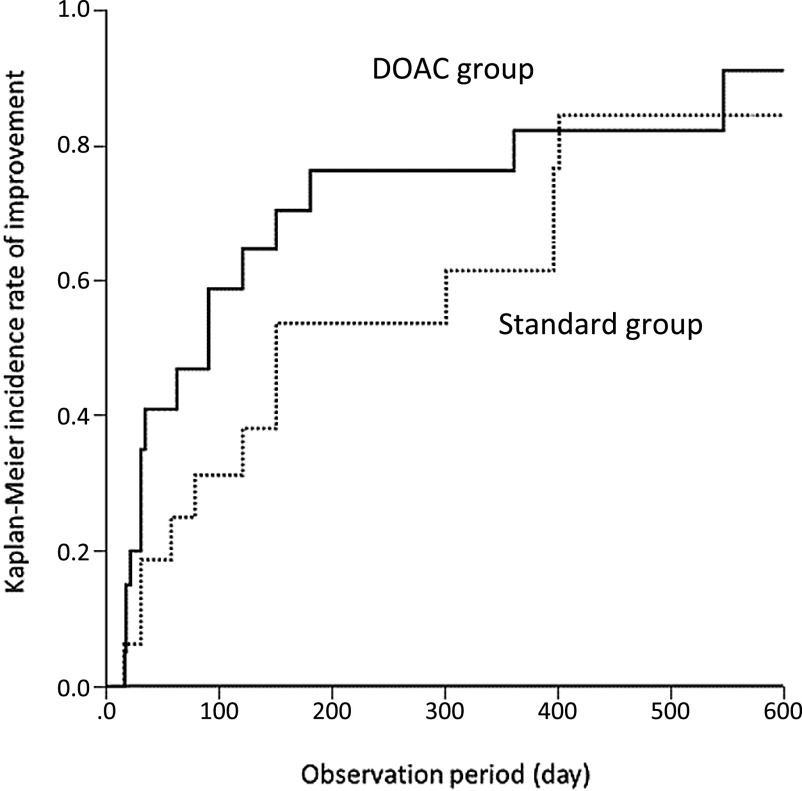
Rates of improvement by Kaplan–Meier analysis The X-axis indicates the observation period (days) and y-axis the rate of improvement. The rates of improvement in the 6 months after starting treatment were 76.4% in the DOAC group and 53.6% in the standard group; this difference this is not significant (log-rank test) (p=0.59).

Improvement in VTE was documented in 17 patients (85%) in the DOAC group and 12 (75%) in the standard group. There was no significant difference in the rate of improvement between the groups 6 months after starting treatment (*p*=0.59, Figure 2). No recurrence of VTE occurred in the DOAC group (0%), whereas two patients (12.5%) had recurrences in the standard group. INR values at the time of recurrence in these two patients were 1.01 and 0.95.

The incidence of adverse events was examined to evaluate safety. Adverse events in the DOAC group comprised epistaxis in one patient (Common Terminology Criteria for Adverse Events [CTCAE^10^] v.4.03, grade 1; Thrombolysis in Myocardial Infarction [TIMI] minimal hemorrhage^11–14^; International Society of Thrombosis and Hemostasis (ISTH)^15^ minor hemorrhage) and anemia in two (CTCAE grade 2). In the standard group, one patient had epistaxis (CTCAE grade 2, TIMI minimal hemorrhage, ISTH minor hemorrhage).

Univariate analysis to evaluate factors influencing the therapeutic outcomes and adverse events showed that chemotherapy significantly impacted improvement in VTE, as shown in Table 2 (hazard ratio: 0.29%; confidence interval [CI]: 0.10–0.78; *p*=0.01).

## Discussion

VTE, a serious condition, is triggered by various factors and can lead to acute and chronic disturbances in pulmonary circulation. In patients with malignant tumors known to be associated with VTE, the incidence of VTE increases two to four times in cancer patients compared to patients without cancer.^16^ The incidence of VTE occurs is particularly high in patients gynecological cancer.^5,6^ Chemotherapy, which along with surgery is the main treatment for malignant tumors, increases the likelihood of VTE. Inpatient treatment, insertion of a central venous catheter, and the presence of inflammation are additional risk factors for VTE.^4,17,18^ Patients with gynecological cancer are at high risk of VTE and VTE is frequently encountered in clinical settings in such patients. The reasons for frequent VTE in gynecological cancer patients are as follows. First, these cancers occur in older patients than other cancers. Second, tumor masses may compress pelvic vessels such as iliac veins. Third, these patients often receive adjuvant chemotherapy, which is a risk factor for VTEs. Furthermore, surgeries for gynecological cancer often require lymph node resection or peritonectomy, which can lead to vascular injury. Vascular injuries also increase the risk of developing VTE.^5,6^ Ligation or clamping of veins frequently results in significant venous intimal wall injury.^19^ The common sites of such injuries are the inferior vena cava, presacral veins, ovarian veins, common and external iliac veins, internal iliac veins, and parametrial and paracervical varicosities.^20^ VTE is the second most common cause of death in cancer patients; additionally, development of VTE is associated with reduced progression-free and overall survival rates and increased rates of recurrence of uterine and ovarian cancer.^1,5,21,22^

As mentioned earlier, though LMVH is mainly used to treat VTE in patients with cancer worldwide, there is no health reimbursement for use of LMWH to treat DVT in Japan. Therefore, UFH and warfarin have traditionally been used to treat VTE and have established efficacy. Unfortunately, traditional VTE treatment can prolong hospitalization because they require intravenous infusions of UFH, increase the risk of recurrence of VTE and of bleeding (during oral warfarin therapy), increase interactions with other drugs and food, and require regular blood tests.^23^ Combining warfarin with an anticancer agent can also result in increased PT-INR and a stronger expression than usual anticoagulant effect.^24^ The use of DOACs, new therapeutic agents for VTE, has therefore been increasing. DOACs exert an anticoagulant action by selectively and directly inhibiting factors Xa and IIa; using DOACs does not require hospitalization because they are oral medications. Other cited benefits include minimal interaction with other drugs and food and not requiring regular blood testing.^9,25,26^

While occasional studies have reported the efficacy of VTE treatment with DOACs in patients with cancer, there are too few of them. LMWH remains the recommended treatment for the management of VTE in patients with cancer.^2,7,27^ Additional data on the efficacy and safety of using DOACs to treat VTE in patients with cancer could influence treatment plans, possibly shortening hospital stays and reducing the number of blood tests required.

In this study, we examined the efficacy and safety of using DOACs in patients with gynecological cancer and VTE. Our findings suggest that DOACs are as effective as standard therapy. There were no significant differences between the two study groups in rates of recurrence of VTE or of adverse events. However, further investigation is needed because this was a small study.

Patients with gynecological cancer are exposed to various risk factors that are associated with development of VTE, including surgery and chemotherapy. VTE is related to the prognosis of the underlying disease, VTE treatment being considered to have important clinical implications in patients with gynecological cancer.^1,5,21,22^ Demonstrating that DOACs have few interactions with other drugs or food and their use can shorten hospital stays should be of great significance to patients with gynecological cancer and gynecologists treating these patients.

This study had some limitations. It was not a randomized controlled trial but a retrospective study conducted in a single institution. The sample size was therefore small. In addition, we assessed changes in the size of thrombi over time by visual assessment of radiological or ultrasound images. For example, we could have defined 25% or more reduction in long diameter of the thrombus as improvement; however, we did not. A large cohort is needed to evaluate the effects of medication using such a scale for analysis. Further large studies are warranted.

## Figures and Tables

**Table1:** Characteristics of the patients and treatments

	Standard group (n=22)	DOAC group (n=21)	*p* value
Characteristics			
Age* (yr)	59.5±13.4	56.8±14.5	0.45
Height* (cm)	156.8±5.5	156.7±5.4	0.95
Weight* (kg)	61.6±14.9	58.7±16.0	0.56
BMI* (kg/m^2^)	25.1±5.7	23.9±6.5	0.58
AST** (IU/L)	19.5 (14.0–25.8)	21.0 (15.5–29.0)	0.54
ALT** (IU/L)	11.0 (8.0–19.3)	11.0 (9.5–20.0)	0.58
D-dimer** (ng/mL)	8.4 (5.4–20.2)	5.9 (2.4–13.8)	0.13
Rate of change of D-dimer**	0.4 (0.06–1.2)	0.6 (0.2–1.6)	0.44
PT-INR**	1.07 (1.12–1.02)	1.02 (1.12–0.98)	0.052
BUN** (mg/dL)	11.4 (7.3–15.5)	11.5 (8.5–17.4)	0.54
Cr** (mg/dL)	0.63 (0.54–0.75)	0.60 (0.52–0.79)	0.75
eGFR* (mL/min/1.73/m^2^)	75.0±26.8	75.8±21.6	0.92
Duration of initial hospitalization** (days)	42.0 (65.0–25.3)	34.0 (68.0–15.5)	0.33
Total duration of hospitalization** (days)	100.0 (38.8–126.0)	75.0 (58.0–123.0)	0.86
Type of VTE: number of patients (%)			
DVT	17 (77.3)	13 (61.9)	0.27
PE	1 (4.5)	0 (0)	0.32
DVT and PE	4 (18.2)	8 (38.1)	0.15
Cancer stage at diagnosis: number of patients (%)			
I	6 (27.3)	8 (38.1)	0.45
II	2 (9.1)	2 (9.5)	0.97
III	8 (36.4)	7 (33.3)	0.84
IV	6 (27.3)	4 (19.0)	0.52
Primary site: number of patients (%)			
Uterine cervix	1 (4.5)	4 (19.0)	0.14
Uterine corpus	10 (45.5)	4 (19.0)	0.065
Ovary/Fallopian tube/Peritoneum	11 (50.0)	14 (66.7)	0.27
Other	2 (9.1)	0 (0)	0.16
Medical comorbidities: number of patients (%)			
Thromboembolism	1 (4.5)	0 (0)	0.32
Hypertension	6 (27.3)	4 (19.0)	0.52
Diabetes	1 (4.5)	0 (0)	0.32
Dyslipidemia	3 (13.6)	4 (19.0)	0.63
Ischemic heart disease	1 (4.5)	0 (0)	0.32
Cancer of other organs	0 (0)	1 (4.8)	0.30
Cerebral infarction	0 (0)	1 (4.8)	0.30
Therapy: number of patients (%)			
Surgical therapy	16 (72.7)	16 (76.2)	0.80
Chemotherapy	17 (77.3)	17 (81.0)	0.77
Radiation therapy	3 (13.6)	3 (14.3)	0.95
Improvement: number of patients (%)	12 (75)	17 (85)	0.59
Recurrence: number of patients (%)	2 (12.5)	0 (0)	—
Adverse events: number of patients (%)	1 (7.7)	2 (15.6)	—

ALT: Alanine aminotransferase AST: Aspartate aminotransferase BMI: Body mass-index BUN: Blood urea nitrogen Cr: Creatinine eGFR: Estimated glomerular filtration rate *mean±SD **median (IQR) IQR: Interquartile range

**Table2:** Predictors by Cox univariate analysis

Variable	Improvement	
	HR (95%CI)	*p* value
Age	0.99 (0.95 to 1.04)	0.80
Height	1.04 (0.94 to 1.15)	0.48
Weight	1.00 (0.96 to 1.03)	0.76
BMI	0.98 (0.90 to 1.07)	0.66
AST	1.05 (0.96 to 1.07)	0.60
ALT	0.99 (0.96 to 1.03)	0.62
D-dimer	1.00 (0.95 to 1.06)	0.94
Rate of change in D-dimer	1.01 (0.99 to 1.03)	0.30
PT-INR	0.38 (0.03 to 4.28)	0.43
BUN	1.06 (0.97 to 1.16)	0.23
Cr*	1.01 (0.74 to 1.38)	0.96
eGFR	0.99 (0.96 to 1.02)	0.50
Warfarin and heparin vs. DOAC	1.31 (0.49 to 3.51)	0.60
DVT	0.67 (0.31 to 1.48)	0.33
PE	—	—
DVT and PE	1.74 (0.78 to 3.88)	0.18
Uterine cervix	—	—
Uterine corpus	0.66 (0.29 to 1.51)	0.33
Ovary/Fallopian tube/Peritoneum	1.23 (0.58 to 2.86)	0.53
Other site	—	—
Thromboembolism	—	—
Hypertension	0.83 (0.33 to 2.07)	0.69
Diabetes	—	—
Dyslipidemia	—	—
Ischemic heart disease	—	—
Cancer of other organs	—	—
Cerebral infarction	—	—
Surgical therapy	0.48 (0.20 to 1.14)	0.10
Chemotherapy	0.29 (0.10 to 0.78)	0.01
Radiation therapy	—	—

ALT: Alanine aminotransferase AST: Aspartate aminotransferase BMI: Body mass-index BUN: Blood urea nitrogen Cr: Creatinine eGFR: Estimated glomerular filtration rate */0.1 mg/dl increase
